# Nutraceuticals in cognitive impairment and Alzheimer’s disease

**DOI:** 10.3389/fphar.2014.00147

**Published:** 2014-06-23

**Authors:** P. Mecocci, C. Tinarelli, R. J. Schulz, M. C. Polidori

**Affiliations:** ^1^Section of Gerontology and Geriatrics, Department of Medicine, University of PerugiaPerugia, Italy; ^2^Geriatrics Department, Medical Faculty, University of CologneCologne, Germany

**Keywords:** cognitive impairment, dementia, Alzheimer’s disease, dietary natural substances, neurodegeneration, nutraceuticals, neuroprotection

## Abstract

Several chemical substances belonging to classes of natural dietary origin display protective properties against some age-related diseases including neurodegenerative ones, particularly Alzheimer’s disease (AD). These compounds, known as nutraceuticals, differ structurally, act therefore at different biochemical and metabolic levels and have shown different types of neuroprotective properties. The aim of this review is to summarize data from observational studies, clinical trials, and randomized clinical trials (RCTs) in humans on the effects of selected nutraceuticals against age-related cognitive impairment and dementia. We report results from studies on flavonoids, some vitamins and other natural substances that have been studied in AD and that might be beneficial for the maintenance of a good cognitive performance. Due to the substantial lack of high-level evidence studies there is no possibility for recommendation of nutraceuticals in dementia-related therapeutic guidelines. Nevertheless, the strong potential for their neuroprotective action warrants further studies in the field.

## INTRODUCTION

Advanced age is often characterized by a decline in a large spectrum of cognitive abilities including reasoning, memory, perceptual speed, and language. The impairment of more of these activities, when lasting long enough and being associated to functional loss is referred to as dementia. Alzheimer’s disease (AD) is the most common and feared form of dementia representing circa 70% of all dementia cases and displaying a dramatic epidemics due to the enormous growth of the aged population worldwide. Incidence of dementia has increased over the recent years although recent epidemiological studies seem to show a decline that needs to be confirmed in the future (Larson et al., 2013). The number of cases is expected to approach a million people per year in USA by 2050 (Alzheimer’s Association, 2009). AD impacts dramatically on everyday life of older adults, being one of the main causes of disability in the old age. There has been an increasing interest in the past decades about interventions that may help to improve cognitive performance in older age or, at least, delay the onset of dementia. Due to the absence of a cure against dementia and AD, the public health priority has focused more recently on prevention of cognitive decline. It is still unclear which factors lead to the molecular cascade of neurodegeneration in AD, but along with genetic and environmental factors vascular pathology and risk factors have been recently shown to play a crucial role in AD pathogenesis (Polidori et al., 2012). Therefore, lifestyle strategies with beneficial effects on neurodegeneration and vascularity – including natural nutrition and nutritional supplementation, cognitive and social activity, physical exercise – have been identified as possible target options for AD prevention (Brown et al., 2010; Polidori et al., 2012; Polidori and Schulz, 2014).

The effect of a correct diet on human health has been reported in many epidemiological studies and randomized controlled trials (RCTs; Everitt et al., 2006). There is clear evidence that a diet rich in specific nutritional food groups (fruit, fish, vegetables) can reduce the incidence and prevalence of some of the main clinical outcomes, such as neurodegenerative disorders, cardiovascular diseases, diabetes, cancer (Grant, 1999; Panza et al., 2004; Sofi et al., 2008; Frisardi et al., 2010). These specific nutritional food groups are rich in micronutrients and vitamins that, for their characteristic of being of nutritional nature and beneficial for health (like pharmaceuticals) at the same time, have been defined as *nutraceuticals* by De Felice in 1989 (Kalra, 2003). Among different types of diet, the Mediterranean pattern obtained a considerable amount of interest in the past recent decades due to the results of large epidemiological and bench studies showing its high content in nutraceuticals. The Mediterranean diet is characterized by a high consumption of plant foods, fish, olive oil as primary sources of monounsaturated fat and moderate intake of wine. This kind of food intake pattern might be particularly healthy due to synergistic actions of its components. Synergistic mechanisms between food components are responsible particularly for the neuroprotective effects displayed by certain nutrients and nutraceuticals. In addition, cardiovascular diseases such as diabetes mellitus, hypertension, and lipid disorders, as well as white substance lesions are highly susceptible to micronutrient changes. This might be particularly important in light of the recently shown major pathophysiological role of vascular pathology and risk factors in both AD and its prodromal phase, mild cognitive impairment (MCI; De la Torre, 2010; Polidori et al., 2012; Féart et al., 2013). Independent of the mechanism through which the Mediterranean dietary pattern exerts its beneficial effects, a large amount of studies have recently shown its protective activity against MCI and AD (systematically reviewed and meta-analyzed by Singh et al., 2013 and Psaltopoulou et al., 2013).

The aim of this review was to present evidence on a particular group of plant and food components, the so-called nutraceuticals, which have displayed over the years the ability or a strong potential to act as neuroprotectans and/or delay cognitive impairment.

Practically, no official definition exists for the term “nutraceutical,” though it is often used to describe a broad list of products sold under the premise of food components with an expressed intent of treatment or prevention of disease and for enhancing the health and wellbeing of an individual (Hardy et al., 2003). In other words, nutraceuticals are foods, or food components, that provide medical or health benefits, including prevention and treatment of several diseases (Calabrese et al., 2009).

In this review, we focused our attention on a group of substances proposed to prevent or treat cognitive impairment or dementia, with a particular attention on AD. Nutraceuticals evaluated in humans in epidemiological, observational, or clinical studies as well as in RCTs were selected. Only molecules with stronger evidence for neuroprotection as well as those more widely studied were included. To avoid redundancies, we refer the reader to our previous studies for the role and use of vitamin E family (Mangialasche et al., 2010, 2012, 2013; Mecocci and Polidori, 2012; Mecocci et al., 2013) and to recent reviews for the role and use of vitamin C (Heo et al., 2013; Harrison et al., 2014) and of omega-3 fatty acids (Lin et al., 2012; Sydenham et al., 2012; Dacks et al., 2013; Denis et al., 2013).

## FLAVONOIDS

Flavonoids are a group of poliphenolic compounds that are very common in the daily human diet. They are found in most plants, including fruits, vegetables, and several types of natural drinks such as tea, cocoa and wine (Manach et al., 2004; Spencer et al., 2008).

On the basis of their chemical structure they can be divided into six subgroups (**Table 1**).

**Table 1:** Flavonoid chemical subgroups and relative food sources.

Groups	Molecules	Food source
FLAVANOLS	Catechin, epicatechin, epigallocathechin, epigallocatechin gallate (EGCG)	Cocoa and chocolate, green tea, grapes
FLAVONOLS	Kaempferol, quercetin	Onions, apples, green tea, capers, leeks, broccoli
FLAVONES	Luteolin, apigenin	Celery, parsley, rosemary
ISOFLAVONES	Daidzein, genistein	Soy
FLAVANONES	Hesperetin, naringenin	Citrus fruit, tomatoes
ANTHOCYANIDINS	Pelargonidin, cyanidine, malvidin	Berry fruits, red wine

Flavonoids and their metabolites modulate several neurological processes as shown by studies in which an interaction with neuronal-glial signaling pathways involved in neuronal survival and function was observed (Spencer, 2010; Williams and Spencer, 2012). In addition, flavonoids induce changes in cerebral blood flow (Francis et al., 2006; Fisher et al., 2006; Williams and Spencer, 2012), upregulate antioxidant enzymes and proteins involved in synaptic plasticity and neuronal repair (Mann et al., 2007; Eggler et al., 2008; Spencer, 2009) and inhibit neuropathological processes in brain regions typically involved in AD pathogenesis (Wang et al., 2008a).

### FLAVANOLS: CATECHIN, EPICATECHIN, EPIGALLOCATECHIN, EPIGALLOCATHECHIN GALLATE

Flavanols are a main flavonoid group and are found in cocoa and chocolate as well as in black and green tea and in grapes. Research over the past decade has identified flavanols as showing diverse beneficial physiologic and antioxidant effects, particularly in the context of vascular function (Francis et al., 2006).

Catechin and epicatechin are the most abundant flavanols in grape seeds and grape juice. A study on supplementation of grape juice from a variety of *Vitis vinifera* called Koshu demonstrated an inhibition of glutamate excitotoxicity (Narita et al., 2011). In humans, few clinical trials with grape juice have shown that short- and moderate-term supplementation produces benefit in individuals with cerebrovascular diseases including increased serum antioxidant capacity and reduced LDL oxidation, improvement of endothelial function and reduction of platelet aggregation (Krikorian et al., 2010a).

Daily EGCG treatment in rats has been demonstrated to reduce the progressive increase of oxidative stress induced by hypertension. EGCG also decreases the concentration of reactive oxygen species on hippocampus. Based on these results a therapeutic effect of EGCG in treating vascular-induced learning and memory impairment has been proposed (Wang et al., 2012). Black and green teas have a high content of catechins, being EGCG the most abundant. There are several studies that show the protective effect of catechins in cellular and animal models of AD. Despite the lack of clinical trials with tea polyphenols in neurodegenerative diseases, epidemiological observations in US and Finnish populations showed a reduced risk of Parkinson’s disease in high consumers of tea (Checkoway et al., 2002; Hu et al., 2007) and a reduced risk of cognitive impairment in a Japanese population drinking green tea (Kuriyama et al., 2006).

Consumption of cocoa flavanols has been previously shown to influence cerebral hemodynamics. It has been suggested that one consequence of the effect of cocoa flavanols on cerebral blood flow might be to improve performance on visual and cognitive tasks as shown after drinking of cocoa beverages (Scholey et al., 2010; Field et al., 2011).

### FLAVONOLS: QUERCETIN, KAEMPFEROL

Quercetin, one of the most studied bioflavonoids, is found in many common foods, such as capers, apples, onions and green tea (Kelsey et al., 2010). Its primary activity is to prevent endothelial apoptosis caused by oxidants, thanks to a highly potent antioxidant activity and cytoprotective effects (Dong et al., 2012). There are several *in vitro* studies about quercetin effects demonstrating that this molecule increases cell survival in neurotoxic conditions as in the presence of hydrogen peroxide (Heo and Lee, 2004), linoleic acid hydroperoxide (Sasaki et al., 2003; Kelsey et al., 2010), interleukin-1β (Sharma et al., 2007; Kelsey et al., 2010). *In vivo* studies have demonstrated that quercetin could have a role in vascular dementia by decreasing the size of ischemic lesions (Dajas et al., 2003; Kelsey et al., 2010). Quercetin improves memory and hippocampal synaptic plasticity in models of impairment induced by chronic lead exposure and it could have a role in neuronal repairing, as shown in spinal cord injury models (Schültke et al., 2003).

Kaempferol is widely distributed in the human daily diet such as fruits, beverages, tea, and vegetables (Aherne and O’Brien, 2002). Kaempferol protects PC12 cells against the oxidative stress induced by H_2_O_2_ (Hong et al., 2009) and improves cognitive learning and memory capability in mice (Lei et al., 2012). It was reported that the intake of flavonols including quercetin, kaempferol, and myricetin has favorable effects on cognitive performance (Spencer, 2009).

Ginkgo biloba (Gb) is a plant whose herbal extracts (mainly EGb761) are often used as an alternative treatment to improve cognitive function. Extracts of Gb include several components, such as the flavonols quercetin and kaempferol as well as terpenoid lactones that are considered to be responsible for the neuroprotective functions of Gb (Rendeiro et al., 2012). Standardized extracts of Gb leaves are studied for their potential to improve memory and cognitive function in general. Hemodynamic, neurotransmitter, and free-radical scavenging effects of Gb have been shown in several studies. All of these biological functions may be relevant to aging and age-related disorders (Maclennan et al., 2002; Brown et al., 2010). For this reason, several *in vivo* studies from humans have been performed and beneficial effects of Gb in prevention and treatment of neurodegenerative disorders like AD have been shown. Improvement of cognitive performance (Le Bars et al., 1997; Kanowski and Hoerr, 2003), memory (Kanowski and Hoerr, 2003), and attention (Le Bars, 2003; Chan et al., 2007) were consistently observed. Two large RCTs on the use of Gb extracts (the GEM and the GuidAge study) did not show less cognitive decline over time in older adults with normal cognition or MCI taking Gb than those assuming placebo (Snitz et al., 2009; Vellas et al., 2012). Also, Gb showed no effects in reducing either the overall incidence rate of dementia or AD in old age individuals with normal cognition or MCI (De Kosky et al., 2008). The latest published Cochrane review including 36 RCTs could not report a significant evidence for a predictable clinical benefit of Gb for people with dementia or cognitive impairment (Birks and Grimley Evans, 2009).

### FLAVONES: LUTEOLIN, APIGENIN

Luteolin is the most abundant flavonoid in plants such as rosemary, celery, and parsley (Chowdhury et al., 2002; El Omri et al., 2012). It has been shown that luteolin has several pharmacological properties including a protective role of DNA against hydrogen peroxide-induced toxicity and anti-inflammatory actions (Cheng et al., 2010; El Omri et al., 2012).

Apigenin was shown to protect neurons against Aβ-mediated toxicity induced by copper, to increase neuronal viability as well as to relieve mitochondrial membrane dissipation and neuronal nuclear condensation (Zhao et al., 2013). Apigenin also modulates GABAergic and glutamatergic transmission in cultured cortical neurons (Losi et al., 2004). Up to now no clinical studies have been performed with luteolin and apigenin in cognitive impairment or AD.

### ISOFLAVONES: SOY - GENISTEIN, DAIDZEIN, GLYCITIN

Soybean (soy) is a rich source of phytoestrogens, especially isoflavones. These are not the only constituents of soy; in fact, soybean contains also several minerals, fibers, proteins and oligosaccharides. The isoflavones from soybean are considered agonists of estrogen receptors. The presence of soy isoflavones may be responsible for the observed memory-improving effects of soybean supplementation. Isoflavones appear to improve cognitive function by mimicking the effects of estrogen, in particular through estrogen receptor β in the brain (Lee et al., 2004a). Estrogen replacement can improve cholinergic function by increasing choline uptake and potassium-stimulated acetylcholine release (Gabor et al., 2003; Bansal and Parle, 2010). Former studies revealed that soy isoflavones improve visual spatial memory and learning ability as well as memory of male and female animals (Duncan et al., 2003; Lee et al., 2004b) and humans (File et al., 2001; Zhao and Brinton, 2007; Bansal and Parle, 2010). Furthermore, soy isoflavones can influence the brain cholinergic system and reduce age-related neuronal loss and cognition decline in male rats (Lee et al., 2004b). A study on young and mature mice (Bansal and Parle, 2010) demonstrated that chronic dietary supplementation with soybean improves cognitive performance, decreases thiobarbituric acid reactive substances (TBARs) and increases plasma glutathione peroxidase levels, suggesting that soy isoflavones have antioxidant properties (Djuric et al., 2001; Lee et al., 2004b).

Few RCTs have been performed with soy supplementation, with controversial results. A long-term supplementation of soy in women had no effect on global cognition but improved visual memory after thirty months (Henderson et al., 2012), while in men, treated for twelve weeks, only spatial working memory improved compared to the placebo group (Thorp et al., 2009). A previous study in postmenopausal women who received soy protein for twelve months had no benefit in cognitive performance (Kreijkamp-Kaspers et al., 2004).

### ANTHOCYANIDINS: PELARGONIDIN, CYANIDINE, MALVIDIN

Blueberry, bilberry, cranberry, elderberry, raspberry seeds, and strawberry are sources of natural anthocyanin antioxidants. Proanthocyanidins extracted from grape seeds (the bark of the Chinese *Scutellaria baicalensis* herb) exert potent anti-inflammatory, antioxidant, antinociceptive, and vasodilatative effects and may show antidepressant properties (Ogle et al., 2013). Berry anthocyanins also improve neuronal and cognitive brain function, ocular health as well as protect genomic DNA integrity (Zafra-Stone et al., 2007). However, blueberries also contain significant amounts of flavanols, flavonols, and other phenolics which may justify their role in increasing their beneficial effects (Harnly et al., 2006).

After blueberries feeding, anthocyanidins are found in specific cerebral sites, including hippocampus and neocortex (Andres-Lacueva et al., 2005). Neurogenesis acting on hippocampus may represent one mechanism by which blueberry flavonoids improve memory. There is strong evidence suggesting that blueberry can improve memory and learning in aged animals. These improvements seem linked to the modulation of important structural and synaptic plasticity markers (Rendeiro et al., 2012). One of the role of anthocyanins in neuroprotection could be mediated through phospholipase A2 inhibition (Frisardi et al., 2010), which is negatively involved in a complex network of signaling pathways linking receptor agonists, oxidants, and proinflammatory cytokines to the release of arachidonic acid and eicosanoid synthesis (Sun et al., 2004).

Memory performance has been demonstrated to be linked to the modulation of the expression of particular proteins like CREB (cAMP-response element-binding protein), which is a pathway known to be activated in response to Aβ and brain-derived neurotrophic factor (BDNF). Changes in CREB and BDNF in berry-feed animals were accompanied by increases in the phosphorylation state of the protein factor ERK, very important for synaptic plasticity and memory formation (Williams et al., 2008). Furthermore, blueberry seems to have a more significant effect on short-term memory than long-term memory, as demonstrated by improved performance in several memory maze tasks (Ramirez et al., 2005; Williams et al., 2008; Rendeiro et al., 2012). Another study (Fuentealba et al., 2011) that underlines the role of berries-extract against Aβ shows how these extracts could partially antagonize two newly found effects of Aβ: the decrease in intracellular Ca2+ activity, an important element in neurodegenerative processes and ATP leakage, an effect of aggregated Aβ (Petrozzi et al., 2007). Short-time blueberry diet might produce benefits on memory in aged rats (Malin et al., 2011) by a suggested alteration of ROS signaling through CREB and MAP-kinase (Brewer et al., 2010). Inflammation pathways and modulation of the expression of inflammatory genes might also be involved (Shukitt-Hale et al., 2008). Finally, there is evidence that anthocyanins have insulin-like and glitazone-like properties which may contribute to improve metabolic function and lipid lowering effects (Kalt et al., 2008; Tsuda, 2008; Krikorian et al., 2010b) as well as to improve memory and reduce depressive symptoms (Krikorian et al., 2010b).

In humans, a prospective evaluation with a food frequency questionnaire showed that a greater intake of blueberries and strawberries is associated with slower rates of cognitive decline in subjects older than seventy years, suggesting the potential protective role of berries on different cognitive functions (Devore et al., 2012).

## NON-FLAVONOID POLYPHENOLS: RESVERATROL AND CURCUMIN

There is evidence that both resveratrol and curcumin, non-flavonoid polyphenols, show beneficial effects in cell culture and *in vivo* models of neurotoxicity and neurodegeneration.

Resveratrol is a polyphenol found enriched in seeds and skin of several fruits, including grapes used for red wine. It is well known mostly for its cardiovascular effects (Bertelli and Das, 2009; Kelsey et al., 2010). Recent evidence has shown that resveratrol can increase 5-HT activity, which could explain its antidepressant properties (Ogle et al., 2013). In animal models it has been shown that resveratrol inhibits noradrenalin and 5-HT reuptake in rats, with increasing hippocampal serotonin (Xu et al., 2010). Resveratrol is also able to reduce inflammation and protect neurons from death, as shown by *in vivo* experiments on animal models of oxidation-induced neuronal toxicity (Alvira et al., 2007; Kelsey et al., 2010). Protection of organotypic hippocampal slices from hydroperoxide insults has been also observed (Karlsson et al., 2000; Kelsey et al., 2010). Other possible mechanisms for the neuroprotective action displayed by resveratrol are related to its antioxidant properties and its capacity of modulation of Aβ processing and up-regulation of the longevity-linked gene sirtuin 1 (Pocernich et al., 2011).

Several studies in humans have shown a lower risk of dementia in subjects drinking moderate amounts of red wine when compared to abstainers (Orgogozo et al., 1997; Truelsen et al., 2002). Furthermore, a small clinical trial in healthy adults showed an increase of cerebral blood flow during cognitive tasks in subjects treated with resveratrol compared to placebo (Kennedy et al., 2010).

Curcumin is the most active element of tumeric (*Curcuma longa*), an herb of the ginger family. It has been a staple of oriental medicine for thousands of years (Ogle et al., 2013). Since the prevalence of AD in Indian countries is much lower than in US, it has been suggested that a diet rich in curcumin may reduce the risk of AD (Ganguli et al., 2000). Curcumin seems to act with different mechanisms including antioxidant, anti-inflammatory and anti-amylodogenic ones. For example, curcumin enhances cell viability by decreasing ROS and inhibiting pro-apoptotic signals in mouse models of encephalitis (Dutta et al., 2009; Kelsey et al., 2010). It also reduces Aβ-related cerebral burden and inflammation in transgenic AD mice (Lim et al., 2001). It has been proposed that curcumin exerts most of its effects by inhibiting monoamine oxidase levels, thereby reducing depression also because it modulates serotonergic, dopaminergic, and noradrenergic transmission (Xu et al., 2012; Ogle et al., 2013).

A 6-month RCT performed in patients with AD showed no beneficial results in cognitive performances (Baum et al., 2008). More recently, a randomized clinical trial with Curcumin C3 Complex -consisting of 95% curcuminoids with 70–80% comprised by curcumin, 15–25% demethoxycurcumin, and 2.5–6.5% bisdemethoxycurcumin-was conducted in mild to moderate AD with no evident benefit on cognitive functions (Ringman et al., 2012).

## CAROTENOIDS

Over 700 different members of the carotenoid family have been identified and about 40 are found in human blood and tissue. Major carotenoids in human organism are lycopene, lutein, zeaxanthin, β-cryptoxanthin, α-carotene, and the most prominent carotenoid, β-carotene. Depending on dietary habits, blood levels vary between 0.01 and 1 mol/L but they can be considerably increased upon supplementation with single compounds or mixtures (Stahl et al., 1992). For structural reasons and based on experimental data carotenoids have been assigned as antioxidants (Krinsky and Johnson, 2005). Carotenoids as natural fat-soluble pigments are found mostly in vegetables and fruits that are red, orange, and deep yellow in color. More recently, astaxanthin, a carotenoid mainly present in seafood, has been extensively studied in *in vitro* and *in vivo* models, showing antioxidant and anti-inflammatory properties as well as protective functions in microcirculation and in mitochondrial functions (Kidd, 2011) suggesting a potential efficacy in several neurodegenerative diseases (Barros et al., 2014). High plasma carotene concentrations associated with lower mortality from all causes were shown by the SENECA investigators (Buijsse et al., 2005), although conflicting data from intervention studies with β-carotene to prevent cancers and cardiovascular disorders have challenged the concept (Polidori and Stahl, 2009). Like other important carotenoids of the antioxidant network, lutein and zeaxanthin, the predominant carotenoids of the macula lutea, are suggested to act as photoprotectants preventing age-related degeneration of the macula lutea (Sabour-Pickett et al., 2012). Astaxanthin showed a protective effect for visual problems, such as blurred vision or reduced visual acuity, and also improved muscle strength and endurance in runners and in soccer players (Kidd, 2011).

Patients with moderate to severe AD showed lower plasma levels of two major carotenoids, lutein, and β-carotene, compared to patients with mild AD or controls (Wang et al., 2008b). Among AD patients a lower MMSE score (Mini Mental State Examination, a measure of cognitive performance) was associated with lower lutein and β-carotene levels in this study (Wang et al., 2008b). Lycopene displays not only sun protective effects (Stahl et al., 2006) but also beneficial effects against development of prostate cancer (Seren et al., 2008). Among six carotenoids tested, lycopene was the only carotenoid inversely associated with quality of cognitive performance as assessed by both MMSE, DemTect (Dementia Detection Test) and Clock Drawing Test in healthy subjects from 45 to 102 years of age (Polidori et al., 2009).

A randomized trial with beta carotene supplementation in men participating to the Physicians’ Health Study showed, after a mean treatment duration of 18 years, better cognitive performances in the treated compared to the placebo group. The same effect was not observed in newly randomized subjects after one year treatment, suggesting that efficacy of beta carotene can be obtained only after a long term supplementation (Grodstein et al., 2007). In a multicenter trial in old age subjects suffering from MCI, a dietary supplement of astaxanthin, phosphatidylserine, and vitamin E improved memory skills after sixty days of treatment, suggesting a significant potential role of his kind of supplementation in counteracting age-related cognitive decline (Zanotta et al., 2014).

## CROCIN – SAFFRON

Crocin is the main chemical compound identified in saffron, whose scientific name is *Crocus sativus*. It has been used over the years in folk medicine as an antispasmodic, gingival sedative, nerve sedative, carminative, and expectorant stimulant (Akhondzadeh and Abbasi, 2006; Akhondzadeh et al., 2010). It has been reported that saffron active constituents have anticonvulsant, antidepressant, and anti-inflammatory effects, and improve learning and memory (Schmidt et al., 2007). Crocin is the actual active component involved in both the improvement of learning and memory and preventing effect of long term potentiation blocked by ethanol (Akhondzadeh and Abbasi, 2006) and its potential in the treatment of neurodegenerative diseases like AD (Khalili and Hamzeh, 2010). In animal models, the effectiveness of crocin has been shown in antagonizing the cognitive deficits caused by neurotoxic agents like streptozocin (Naghizadeh et al., 2013).

Crocin improved cognition as evaluated by means of ADAS-Cog and CDR-SB in subjects with mild to moderate AD (Akhondzadeh et al., 2010). In a recent *in vivo* study, it has been demonstrated that crocin significantly modulate the levels of oxidative markers in the hippocampus, abolishing the deleterious effects of chronic stress on learning and memory (Ghadrdoost et al., 2011).

## B-VITAMINS: FOLATE, COBALAMIN, PYRIDOXIN

Folate (vitamin B9), cobalamin (vitamin B12), and pyridoxin (vitamin B6) are the most studied B-vitamins in the field of cognitive decline and dementia (Brown et al., 2010). They are essential for maintaining the integrity of the nervous and hematopoietic systems (Malouf and Areosa Sastre, 2009).

Folate is absorbed from the diet and its decrease in blood is highly dependent on a poor diet, malabsorption, and alcoholism; cobalamin deficiency is also associated with malabsorption due to digestive disorders occurring in older adults, and can result in irreversible neurological disorders such as peripheral neuropathy (Rébeillé et al., 2007; Clarke, 2008). Vitamin B6 - comprising three chemically distinct compounds, pyridoxal, pyridoxamine, and pyridoxine - is involved in the regulation of mental function and mood. It is also an essential homocysteine re-methylation cofactor, and its deficiency is associated with an increase in blood homocysteine levels (Malouf and Grimley Evans, 2003). Poor vitamin B6 status is common among older people. Folate and cobalamine deficiencies cause accumulation of homocysteine. Many studies have found cross-sectional associations between low circulating folate levels or hyperhomocysteinemia and low MMSE scores (Morris and Jacques, 2009; Morris et al., 2012). Vitamin B12 is involved in the methylation of homocysteine to methionine for the synthesis of methyl acceptors such as membrane phospholipids, myelin and neurotransmitters. Homocysteine is potentially toxic to neurons, its levels have been associated with atrophic changes in the brain and are negatively correlated with neuropsychological tests scores; it is also considered a marker for low serum vitamin B12 and folate (Ellinson et al., 2004).

A prospective study over a 4, 5-year period found that homocysteine is a risk factor for dementia or cognitive impairment (Haan et al., 2007). In this study, plasma cobalamin levels were associated with reduced risk (Haan et al., 2007). A recent clinical cohort trial found that plasma homocysteine levels are inversely related to cognitive performance, but no evidence of a significant protection of high plasma folate against dementia was found (Morris et al., 2012). However, B12- and B6-vitamin treatment has been demonstrated to stabilize performance on the CLOX test (Royall et al., 1998) as well as on executive and planning function (De Jager et al., 2012). Kado et al. (2005) and colleagues demonstrated that folate, but not cobalamin levels, are independently predictive of cognitive decline over a 7-year period in high functioning old people.

These heterogeneous and contradictory results are reported in the last Cochrane reviews, in which a significant effect of B-vitamin treatment in cognitive function could overall not be reported (Malouf and Grimley Evans, 2008; Malouf and Areosa Sastre, 2009).

Supplementation with B vitamins including vitamin B6 has been shown to reduce blood homocysteine levels. In addition, B6 vitamin concentration has been associated with better global cognition scores, especially with better attention, and executive function scores (Moorthy et al., 2012). Although vitamin B6 did not succeed in reducing atherosclerotic manifestations in hyperhomocysteinemic patients (Cacciapuoti, 2013), neuropsychiatric disorders - seizures, migraine, chronic pain, and depression - have been linked to vitamin B6 deficiency. However, there is no evidence that short-term treatment with vitamin B6 improves mood (depression, fatigue, and tension symptoms) or cognition (Malouf and Grimley Evans, 2003).

Recent RCTs on the effects of folate, vitamin B12, and vitamin B6 supplementation have been performed in subjects with mild to moderate AD. These failed in showing any effect of supplementation in slowing cognitive decline (Sun et al., 2007; Aisen et al., 2008; Malouf and Grimley Evans, 2008; Malouf and Areosa Sastre, 2009). A review on supplementation of vitamins B12, B6, and folic acid alone or in combination showed that results from nineteen RCTs did not appear to improve cognitive function in individuals with or without existing cognitive impairment (Ford and Almeida, 2012). So, it remains to be established if prolonged treatment with B-vitamins can reduce the risk of dementia in later life. However, in a recent study high-dose B-vitamin treatment (folic acid, vitamin B6, and vitamin B12) not only slowed shrinkage of the whole brain volume over 2 years but it reduced, by as much as sevenfold, the cerebral atrophy in those gray matter regions specifically vulnerable to the AD process, including the medial temporal lobe (Douaud et al., 2013).

## DITERPENES: CARNOSIC ACID, AND ROSMARINIC ACID

Carnosic and rosmarinic acids are two of the most important antioxidant compounds in rosemary. They are parts of the bigger family of phenolic acids and diterpenes, that have been extensively studied (Yang et al., 2001; Kayashima and Matsubara, 2012). Both carnosic and rosmarinic acid showed a neuroprotective action both in *in vitro* models of neuronal death and in *in vivo* models of neurodegeneration. They scavenge reactive nitrogen species (Qiao et al., 2005; Kelsey et al., 2010) and protect neuroblastoma cells from hydrogen peroxide-induced oxidative stress (Lee et al., 2008; Kelsey et al., 2010).

Diterpenes also significantly alleviate memory impairment associated with Aβ neurotoxicity in AD and significantly delay the onset of the disease (Kelsey et al., 2010; Shimojo et al., 2010). Recently it has been found that carnosic acid has antiangiogenic effects (Kayashima and Matsubara, 2012). The neuroprotective effects of this substance, therefore, might be explained on the basis of the recently identified role of angiogenesis in the formation of β-amyloid plaques and the consequent neurotoxicity (Vagnucci and Li, 2003). The exact mechanism by which carnosinic acid inhibits angiogenesis is not clear; however, its antioxidant activity seems to play an important antiangiogenic role.

## CONCLUSION AND FUTURE PERSPECTIVE

When placed in the context of a healthy lifestyle behavior, age-related changes in nutrition may play an important role in brain functioning as well as in major organ functioning of old people. Susceptibility of elderly population to specific nutrient deficits may exacerbate processes of cognitive decline. Indeed, there is large evidence regarding benefits of several nutrients in the common diet towards cognitive impairment and other diseases. There is great public and scientific interest about the potential of nutritional supplementation to prevent age-related diseases in general and cognitive decline in particular by counteracting deleterious neurodegenerative and pathological processes. We reviewed several components of common diets and several phytochemicals that have been shown to have benefits on these diseases and cognitive impairment. Unfortunately, there is a substantial lack of well conducted studies to be included in comparison analyses; scientific literature is still poor of RCTs (a summary is reported in **Table 2**) for effects of some of these molecules, and lots of studies are conducted on either *in vitro* models or animal models. A very few studies testing the effects of a combination of two substances or antioxidant mixtures also display little benefit against AD onset or progression as well as against the transition of MCI to AD. All critical components of studies on nutraceuticals in cognitive impairment, from sample size to cell types, to dosage to cognitive measures used, are not comparable. For instance, diagnostic criteria of MCI and AD as well as inclusion criteria and outcomes are not homogeneous. Start and end of the intervention with a particular nutraceutical in MCI or AD have been set for logistic reasons mostly so that intervention begin occurs too late during the course of the disease and study end delimits a far too short intervention period. Finally, specific compound-related issues might be responsible for the lack of clear success of intervention, including dosage-, kinetic-, bioavailability-, metabolic, and genetic-related issues that have been repeatedly discussed (http://lpi.oregonstate.edu/infocenter/cognition.html; Polidori and Schulz, 2014). Despite this, it seems difficult to uniform trial design and method in AD nutritional studies.

**Table 2:** Nutraceuticals in clinical trials: reference, dosage, and study quality (*low; **moderate; ***high; ****very high).

	References	Dosage	Quality
**Flavanols**
Catechin, epicatechin, epigallocatechin, EGCG	Krikorian et al. (2010a), Field et al. (2011), Scholey et al. (2010)	6–9 ml/kg of grape juice, 720 mg of cocoa flavanols, 300 mg EGCG	*, **, **
**Flavonols**
Quercetin-kaempferol in Gingko biloba	Birks and Grimley Evans (2009; Cochrane)	80–720 mg /day of EGb761 (Gb extract)	****
**Isoflavones**
Genistein, daidzein, glycetin in soy	Kreijkamp-Kaspers et al. (2004), Thorp et al. (2009), Henderson et al. (2012)	25.6 gr /day of soy protein, 116 mg/day isoflavone equivalent, 25 mg/day of soy protein	***, **, ***
**Anthocyanidins**
Pelargonidin, cyanidine, malvidin in berries	Krikorian et al. (2010b), Devore et al. (2012)	6–9 ml/kg of blueberry juice, dietary intake of berries	*, ***
Resveratrol, curcumin	Kennedy et al. (2010), Ringman et al. (2012)	250 or 500 mg resveratrol, Curcumin C3 Complex 2–4 gr/day	*, **
**Carotenoids**
Beta carotene, astaxanthin	Grodstein et al. (2007), Zanotta et al. (2014)	50 mg/alternate days, extract of astaxanthin	***, **
Crocin	Akhondzadeh et al. (2010)	30 mg/day saffron	**
Vitamin B6, vitamin B12, folic acid ±vit. B12, folic acid, vit.B12, vit. B6	Malouf and Grimley Evans (2003) (Cochrane), Malouf and Areosa Sastre (2009) (Cochrane), Malouf and Grimley Evans (2008) (Cochrane), Ford and Almeida (2012)	See reviewed studies	**, **, ***, ***

*For the Cochrane reviews the quality refers to the mean quality of the reported studies.*

Theoretically, phytochemical-based treatments for geriatric cognitive decline and depression could be moved into a stronger clinical trial level on humans, especially due to their low toxicity and high bioavailability. Future studies addressing whether short-term or long-term dietary intake of nutraceuticals can reduce the severity and incidence of neurodegenerative and other age-related diseases appear critical and important for the future.

## Conflict of Interest Statement

The authors declare that the research was conducted in the absence of any commercial or financial relationships that could be construed as a potential conflict of interest.
